# Patient decision-making about complementary and alternative medicine in cancer management: context and process

**DOI:** 10.3747/co.v15i0.280

**Published:** 2008-08

**Authors:** L.G. Balneaves, L. Weeks, D. Seely

**Affiliations:** * School of Nursing, University of British Columbia, Vancouver, BC.; † Department of Community Health Sciences, University of Calgary, AB.; ‡ Department of Research and Clinical Epidemiology, The Canadian College of Naturopathic Medicine, Toronto, ON.

**Keywords:** Complementary therapies, decision-making process, oncology, health professionals, communication

## Abstract

**Objective:**

In this paper, we set out to describe the personal and social contexts of treatment decisions made by cancer patients concerning complementary and alternative medicine (cam) and also the process through which cancer patients reach cam decisions throughout the cancer trajectory.

**Methods:**

We selected and reviewed a variety of cam decision-making models published in the past 10 years within the Canadian health literature.

**Results:**

The cam decision-making process is influenced by a variety of sociodemographic, disease-related, psychological, and social factors. We reviewed four main phases of the cam decision-making process:

- Taking stock of treatment options
- Gathering and evaluating cam information
- Making cam decisions
- Revisiting the cam decision

Immediately following diagnosis, cancer patients become interested in taking stock of the full spectrum of conventional and cam treatment options that may enhance the effectiveness of their treatment and mediate potential side effects. Information about cam is then gathered from numerous information sources that vary in terms of credibility and scientific legitimacy, and is evaluated. When making a decision regarding cam options, patients attempt to make sense of the diverse information obtained, while acknowledging their beliefs and values. The cam decision is often revisited at key milestones, such as the end of conventional treatment and when additional information about disease, prognosis, and treatment is obtained.

**Conclusions:**

The cam decision-making process is a dynamic and iterative process that is influenced by a complex array of personal and social factors. Oncology health professionals need to be prepared to offer decision support related to cam throughout the cancer trajectory.

## 1. INTRODUCTION

Since the late 1990s, a growing number of studies have focused on the engagement of cancer patients in the treatment decision-making process 1–4. The use of qualitative research methods has provided a greater understanding of how cancer patients make sense of the treatment recommendations provided by their health professionals, and of the process by which they seek additional information and evaluate the risks and benefits of available treatment options 5–8. Individuals living with cancer have varying needs regarding their preferred level of involvement in treatment decisions, and correspondingly, vary in the way in which they can be best supported by health professionals during the decision-making process 9,10. Although previous research has provided insight into the decision-support strategies required by patients faced with conventional treatment decisions, its applicability to patients making decisions about complementary and alternative medicine (cam) is questionable.

The literature suggests that most Canadian cancer patients use cam at some point during their illness 11,12. It is therefore essential that oncology health professionals understand and acknowledge the unique contexts and processes that influence treatment decisions specific to cam for each patient. This acknowledgment is especially important given the emerging field of integrative oncology in North America, in which evidence-based cam therapies are gradually being integrated into mainstream cancer care 13. Decision-support strategies that facilitate patients’ informed use of cam and full disclosure of cam use with health professionals are essential for the safe integration of cam with conventional cancer care.

Canadian researchers have taken a leading role in examining the treatment decision-making process of cancer patients interested in cam 14–18. This work has resulted in the development of several decision-making models that capture the complex interplay between key personal, social, and cultural factors and the cognitive processes that underlie the cam decision-making process. Although these models have been limited mainly to breast and prostate cancer and have yet to be empirically tested, they provide health professionals with insight into the experiences of patients making cam decisions, and they highlight moments during the cancer trajectory when patients may possibly benefit most from decision support.

**FIGURE 1:**
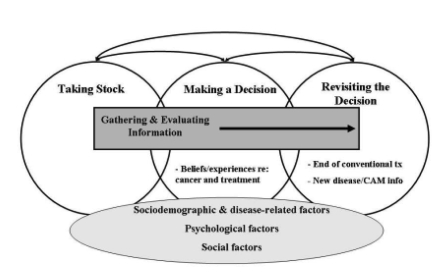
The complementary and alternative medicine (cam) decision-making process.

In the present paper, we begin our discussion by highlighting the highly complex, dynamic, and individualized nature of cam decisions, which results from the unique personal, social, and cultural contexts in which these decisions are embedded. We then draw on previous decision-making models and provide a summary of the main stages within the cam decision-making process (Figure 1):

- Taking stock of treatment options
- Gathering and evaluating cam information
- Making a decision
- Revisiting the decision

This overview emphasizes the iterative nature of the cam decision-making process and how it unfolds across the cancer trajectory.

## 2. DISCUSSION

### 2.1 The Context of CAM Decisions

The decision about whether to use cam, and if so, the type or types of cam to use, is influenced throughout the cancer trajectory by a myriad of factors that can be grouped as follows: sociodemographic and disease-related factors, psychological factors, and social factors.

#### 2.1.1 Sociodemographic and Disease-Related Factors

At a basic level, specific demographic and disease-related factors have been found to be associated with cam use, including age (younger), sex (women), and socio-economic status (higher education and income) 19–21. These personal factors have been identified as being predictive of other self-care health behaviours and reflect health care access issues and the increased use of health care services by women 22. Further, despite an increasing number of private health insurance plans providing coverage for specific cam practices (such as acupuncture and chiropractic treatment), many cam therapies are not covered through public or private health insurance. As a result, the decision to use cam can be an expensive undertaking for many patients, particularly those who are on disability as a result of their illness.

In Canada’s multicultural society, it is also imperative to acknowledge the important influence that ethnicity may have on cam use. For a growing number of immigrants and indigenous peoples, traditional medical systems (for example, Traditional Chinese Medicine, Ayurveda, First Nations traditional healing) are the primary source of health care. As a result, many cancer patients arrive at initial consultations already using or interested in trying cam therapies that are not considered “alternative” within their ethnocultural community.

Increased cam use has also been related to disease characteristics. It has been observed to be higher in breast and prostate cancer populations than in populations with other cancer diagnoses 20,23. The heightened interest in cam in these populations may be a consequence of the proactive nature of these patient groups with regard to advocacy and self-care (that is, support-group membership). Cancer patients with advanced disease have also been found to have a heightened interest in cam 24, which may reflect their attempt to maintain hope when presented with a poor prognosis and limited conventional treatment options, coupled with a search for healing when cure is not possible. Lastly, cam therapies that require intensive time and energy, such as restrictive diets or frequent visits to a practitioner, may be impractical for patients undergoing active cancer treatment, particularly for those experiencing fatigue or other debilitating physical or psychological symptoms.

#### 2.1.2 Psychological Factors

Equally influential in the cam decision-making process are psychological factors. For many individuals, the initial decision to explore cam treatment options arises from a strong internal locus of control (that is, the tendency to attribute event outcomes to one’s own control 25) and a desire to be an active participant in treatment decisions 18,26–29. In addition, for some patients, cam therapies may also provide the hope and optimism required to cope with the cancer journey 18,30. For others, their fears about death and dying may motivate their search for treatment options beyond conventional cancer care so that they can “cover all their bases” 18,31.

#### 2.1.3 Social Factors

It is important to recognize how the personal and psychological factors associated with cam use are also embedded within a larger social context that legitimizes and reinforces the exploration and use of many cam therapies. For example, the increasing tendency of cancer patients to consider cam as a treatment option during their illness may reflect the currently persistent postmodern ideals of individualism, consumerism, and holism 32,33. In addition, an individual’s understandings of what constitutes appropriate treatment and how it can best be achieved are derived not only from personal experience, but also from social interaction and interface with cultural products—most notably the mass media 32. Information about cam is increasingly available and accessible through media sources 34, which lend visibility and perceived legitimacy to this group of therapies and practices.

Further, male and female cancer patients both describe family members, friends, and fellow cancer survivors to be highly influential in their cam decisions 15,16,35,36. Members of a patient’s social network can take on a variety of decision-support roles depending on a patient’s diagnosis and stage of illness, and the nature of their relationship with the patient 35,36. These roles include “interested bystander” (a person who listens and supports a patient’s cam decisions), “information gatherer and reviewer” (a person who helps collect and evaluate information), and “director” (a person who takes over the decision-making process on behalf of the patient) 36.

### 2.2 The CAM Decision-Making Process

Unlike the many rational treatment decision-making models presented within the health care literature, the cam decision-making process has been described as a dynamic and iterative process that is highly variable across individuals 14,15,18. Despite its complex, nonlinear, and individualized nature, some common stages of the cam decision-making process can be explicated.

#### 2.2.1 Taking Stock of Treatment Options

Research has shown that the cam decision-making process begins immediately following a diagnosis of cancer 14,15,18. Despite being emotionally overwhelmed by the news of their diagnosis, most cancer patients are eager to learn about the full spectrum of treatment options and often do not distinguish between conventional and complementary therapies 18. At the time of diagnosis, patients are particularly interested in cam therapies that will enhance the effectiveness of their conventional treatment protocols and mediate potential side effects 14 such as fatigue, nausea and vomiting, and anxiety. However, the already complex decision about whether to use cam is challenged by concerns held by some patients and their health professionals regarding the potential risks posed by inappropriate cam use 14,16,37.

#### 2.2.2 Gathering and Evaluating CAM Information

During the initial phase of taking stock of available treatment options and identifying a personal interest in cam, cancer patients begin to gather and evaluate information about the possible role of cam in their cancer experience. Because the decision-making process is highly dependent on the patient’s unique contextual factors, the process of gathering and evaluating information is highly variable across individuals.

For some cancer patients, particularly those who feel overwhelmed by their diagnosis, the information gathering and evaluation phase is a passive process in which they seek information only about the cam therapies that they have had previous experience with or that are recommended by a trusted health professional, family member, or friend 14,15,18. Other cancer patients take on a more active role in which they engage in an extensive and iterative information-seeking and evaluation process related to a diverse range of cam therapies. This process often continues throughout their cancer journey and is revisited at key milestones, such as at the end of conventional cancer treatment and at diagnosis of recurrence 14,18,35. For these individuals, the search for cam information is motivated by their information needs, including the potential risks and benefits of cam use, the likelihood of negative interactions of specific cam therapies (typically natural health products) with conventional treatments (that is, chemotherapy, radiation, hormone therapy), the appropriate timing of cam use in the conventional cancer treatment trajectory, and the availability and financial cost of specific therapies 14,15,18.

The type of evidence privileged by cancer patients in making cam decisions varies widely and includes professional advice, the scientific literature, anecdotes about cam use from social networks, the Internet, and previous personal experiences with cam 14,35,38. As a result, patients seek information about cam from a multitude of sources, although there is a preference to seek assistance from trusted individuals who are perceived as being credible, such as oncologists, family physicians, or regulated cam practitioners (a naturopathic physician, for instance) 14,16,39. Whether these health professionals have the training, knowledge, or interest to discuss cam therapy options with cancer patients, however, is discussed in elsewhere in this issue 40.

The information gathering and evaluation phase can be an anxiety-provoking experience. Some individuals are able to control the amount of information they obtain about cam by restricting their search to a limited number of therapies or by avoiding certain resources, such as the Internet, but others report feeling overwhelmed by the amount of cam information they acquire 14,18. Some patients struggle to make sense of the often contradictory information that exists about cam 14,41, and they report being particularly distressed about the lack of consensus between and among their cam and conventional health professionals about the implications of cam use. This conflict causes profound anxiety in some cancer patients who are fearful of making the “wrong” treatment decision that could have potentially serious consequences regarding their survival.

#### 2.2.3 Making a CAM Decision

How cancer patients ultimately reach a decision about cam varies considerably between individuals and along the cancer trajectory. This complex process, labelled “bridging the gap” by Balneaves *et al.* 14, involves cancer patients attempting to make sense of the disparate advice and information they have gathered about cam while reflecting on their personal beliefs about cancer, treatment, and healing.

In our previous work, we identified three different types of cam decisions.

First, individuals in the midst of conventional treatment who are experiencing high anxiety and conflict often “take it one step at a time” and postpone their cam decisions to later in the cancer trajectory when they have more energy to reflect on a broader spectrum of treatments. This delay is particularly evident in patients who have received limited support from their oncology health professionals in the cam decision-making process. The cam therapies chosen by these patients are typically those that fall within the realm of supportive care (for example, massage, relaxation therapy) and have been associated with positive psychosocial outcomes 14,18.

Second, cancer patients who have a high level of trust in the conventional health care system engage in a “playing it safe” decision-making process in which the advice of their oncologists is privileged throughout the cancer trajectory. Only cam therapies that can be easily incorporated into their conventional treatment protocol are chosen. Frequently, these patients perceive their cancer diagnosis to be “too serious to play around with” and are hesitant to use any therapies, particularly natural health products, that may negatively interact with their conventional treatment 14,42.

In contrast, a third group of cancer patients is able to “bring it all together” and make treatment decisions that incorporate cam as part of their treatment plan with minimal conflict and anxiety. These individuals often report having a life-long commitment to cam use that precedes their diagnosis, and they believe that cam therapies are natural, supportive of the body’s innate ability to heal, and better able to holistically address physiologic and psychosocial needs than conventional care can 14,35,43. However, some of these patients have described feeling “pushed” towards cam as result of their beliefs about conventional cancer treatments being “toxic,” “poisonous,” or immunosuppressive 21,37. Still others have turned toward cam because of their dissatisfaction with conventional care, including the quality and quantity of their interactions with health professionals, the adverse effects of conventional treatment, and their experiences with ineffective therapies 16,35,37,44,45. These latter patients are most at risk of abandoning conventional care in lieu of alternative treatments.

#### 2.2.4 Revisiting the CAM Decision

As patients move through the cancer trajectory and reach the end of their conventional treatment protocol, many revisit the cam decisions made following their diagnosis and during adjuvant treatment. For some individuals, this reflection is a consequence of feeling as if they have “fallen off the cliff” as they lose the frequent contact they have had with their oncology health professionals during active treatment 46. Adding cam therapies to their health care repertoire allows these individuals to feel as if they are “still doing something” and helping their bodies recover from the trauma of chemotherapy and radiation. Other cancer patients see the end of conventional treatment as opening the door to specific cam therapies, especially natural health products, which were discouraged while they were undergoing treatment because of fears of negative interactions 14. Lastly, the completion of conventional treatment also liberates patients from what can be an extensive commitment of time and physical and emotional energy; it gives them the opportunity to explore new treatment options that were too overwhelming to consider at the beginning of the illness experience.

Cancer patients also revisit their cam decisions in response to new information received regarding disease progression and prognosis 14,18. The identification of a recurrence or metastases can result in some individuals returning to the taking-stock phase of the decision-making process to reconsider their cam decisions and perhaps expand their search for cam therapies, including more alternative forms of treatment. Others interpret disease progression as a sign of ineffectiveness, and they withdraw or significantly alter their cam regimen. Conversely, results suggestive of remission or tumour regression can encourage some individuals to maintain or increase their use of cam.

Lastly, the growing field of cam research is providing new data on the efficacy and safety of specific cam therapies on almost a daily basis. This information is rapidly translated to cancer patients and oncology health professionals through the media, scientific journals, and research-based databases [for example, Natural Standards (www.naturalstandard.com)]. For individuals for whom scientific evidence is an important consideration in their treatment decisions, such information may encourage their exploration of promising cam therapies or their withdrawal from products or practices suggested to be ineffective or potentially harmful 14,38.

## 3. SUMMARY

Our review of the cam decision-making process (see Figure 1) highlights the fact that cam decisions are highly individualized, complicated, and multifaceted, and that they involve dynamic processes that vary throughout the cancer trajectory. The decision to use—or not to use—cam is not a one-time whimsical decision; instead, it is a decision that leads cancer patients to reflect on their unique personal and social context and to ponder how cam may fit with their values, beliefs, and specific health care needs. As the individual and social contexts of patients change, the appropriateness of select cam therapies in their treatment regimen also changes. Decisions about cam are not static; rather, they are dynamic entities that require assessment and follow-up by health professionals throughout a patient’s illness.

Recognizing that cam decision-making can be an anxiety-laden experience for patients suggests the need for ongoing decision and information support by oncology health professionals from diagnosis through survivorship—and for some individuals, through end of life. Research indicates that cam decisions coincide with patients’ decisions about conventional treatment, with many individuals regarding conventional and cam therapies as part of the same continuum of care 14,18. As a consequence, cancer patients often assume that discussions about treatment options with their oncology health professionals will include both conventional and cam therapies. However, oncology health professionals are often hampered in these types of discussions because of the limited evidence that supports the safety and efficacy of many cam therapies in the context of conventional cancer care and because of the lack of integration of cam education into their professional training programs 47,48.

Despite the need for additional research and professional education on cam, it is essential that oncology health professionals begin a dialogue with patients that focuses not just on individual cam therapies, but also on how to make treatment decisions that acknowledge scientific evidence and each patient’s beliefs, values, and sociocultural contexts.

A dialogue regarding cam-related treatment decisions would ideally be an ongoing discussion that corresponds with key milestones in a patient’s illness, such as diagnosis, end of conventional treatment, and recurrence or metastasis. A key element of patient-centered decision support is an assessment of an individual’s preferred level of engagement in the decision-making process and preferred degree of involvement of their oncology health professionals. Further, recognizing the individual contexts of patients, including pre-existing beliefs about healing, previous experiences with conventional medicine and cam, and the influence of significant others and the surrounding community, is also essential. Assessing patient goals related to cam use is also important and can illuminate important belief systems and experiences that need to be acknowledged in tailoring decision support. Although some patients may be interested in using cam for curative reasons and to address the side effects of conventional treatment, other patients may be pursuing cam to preserve hope in the face of a terminal prognosis. In addition, acknowledging the potential barriers to cam use can help patients consider the fuller implications of using cam therapies beyond the physiologic effect and their own physical limitations in using a diverse range of therapies.

Verhoef, Boon, and Page 40 provide further suggestions regarding how oncology health professionals can communicate with patients about cam. As research in this field continues, we can expect to see evidence-based decision-support strategies emerge, such as decision-support aids and counselling strategies that will assist patients and professionals alike in the cam decision-making process.
